# Supplementary material for: Development of a Mobile Device for Odor Identification and Optimization of Its Measurement Protocol Based on the Free-Hand Measurement
Source: Sensors (Basel). 2020 Oct 30;20(21):6190. doi: 10.3390/s20216190 (PMC7663627; doi:10.3390/s20216190)
Supplement: Supplementary file 1 [file sensors-20-06190-s001.pdf]

sensors

MDPI

Supplementary Materials

Transfer Function Ratios (TFRs)

TFRs calculated from the measurement data obtained through Protocol-5 are shown in the following figures. Mean values and standard deviations of $\log|K_{m,n}|$ and $\arg K_{m,n}$ are plotted as squares and shaded areas, respectively. TFRs for water are also shown in Figure 2 in the main text.

Water

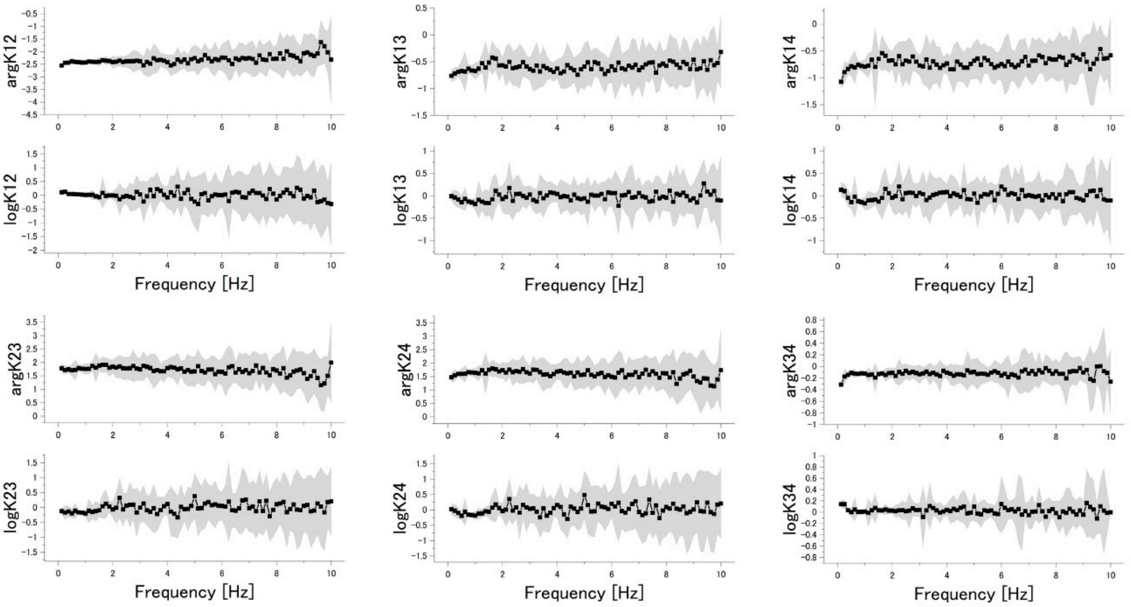

Hexane

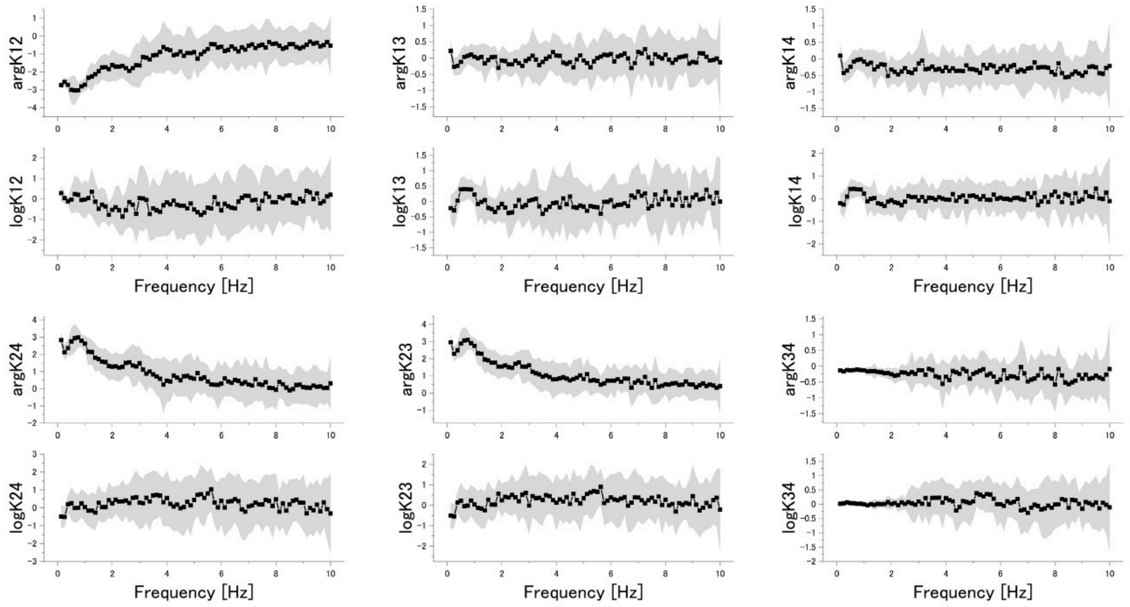

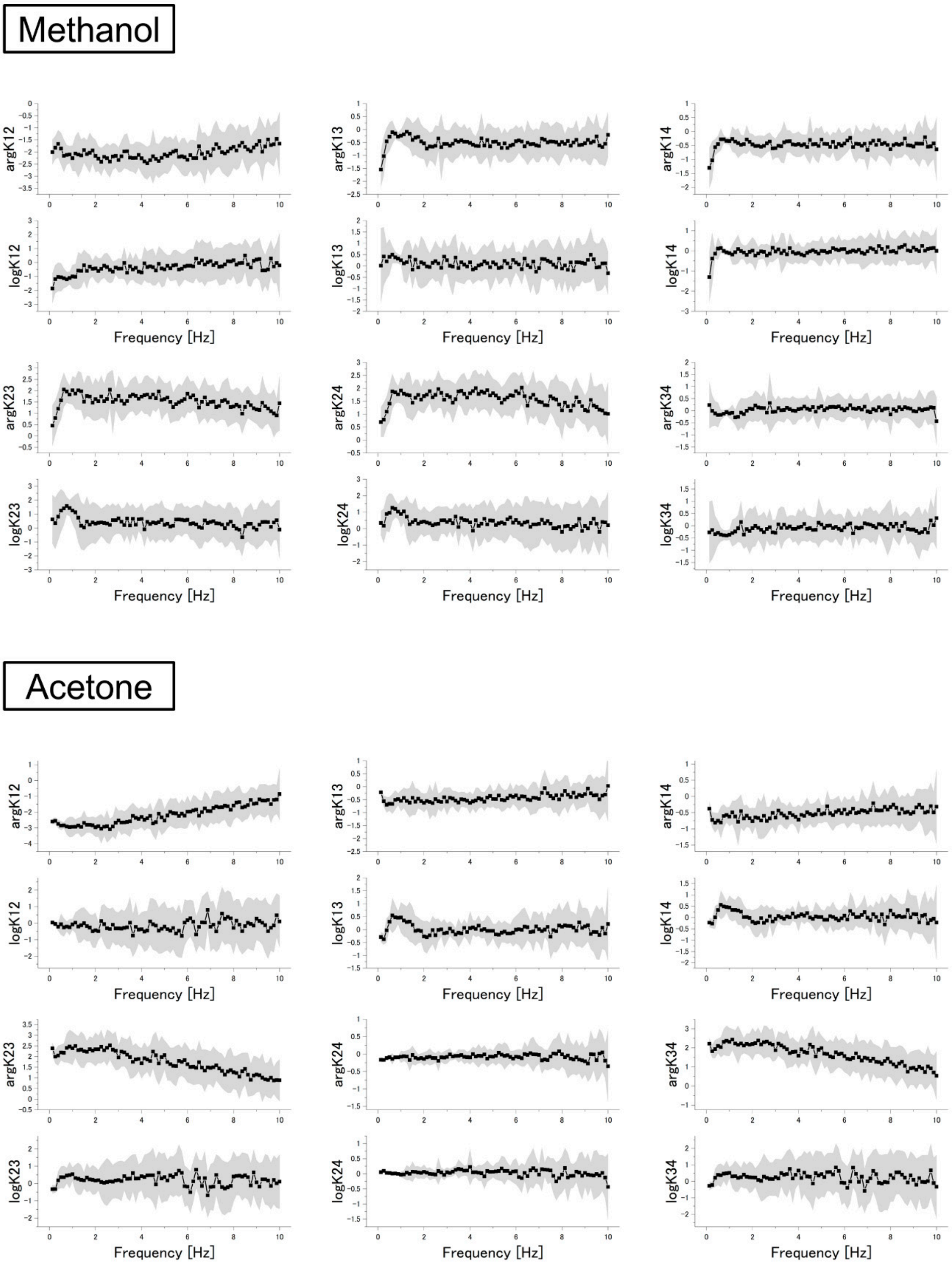

Figure S1. TFRs of the data obtained through Protocol-5.
